# Acute Pancreatitis Increases the Risk of Gastrointestinal Cancer in Type 2 Diabetic Patients: A Korean Nationwide Cohort Study

**DOI:** 10.3390/cancers14225696

**Published:** 2022-11-20

**Authors:** Jin Ho Choi, Woo Hyun Paik, Dong Kee Jang, Min Kyu Kim, Ji Kon Ryu, Yong-Tae Kim, Kyungdo Han, Sang Hyub Lee

**Affiliations:** 1 Department of Internal Medicine and Liver Research Institute, Seoul National University Hospital, Seoul National University College of Medicine, Seoul 03080, Republic of Korea; 2 Department of Internal Medicine, Seoul Metropolitan Government Boramae Medical Center, Seoul National University College of Medicine, Seoul 07061, Republic of Korea; 3 Department of Statistics and Actuarial Science, Soongsil University, Seoul 06978, Republic of Korea

**Keywords:** acute pancreatitis, diabetes mellitus, gastrointestinal cancer, nationwide cohort study

## Abstract

**Simple Summary:**

The effect of acute pancreatitis on diabetic patients in terms of the occurrence of malignant tumors is not well understood. The main contribution of this study is to investigate the association between acute pancreatitis and gastrointestinal cancer in type 2 diabetic patients. Diabetic patients who had a history of acute pancreatitis showed a higher incidence of all gastrointestinal cancers, not only pancreatic cancer. The risk of gastrointestinal cancer in diabetic patients was increased by 1.6 to 4.5 times depending on the history of acute pancreatitis. It seems necessary to investigate the history of acute pancreatitis in diabetic patients and more actively recommend screening for gastrointestinal cancers in such patients. Results of this study suggest that proper management or prevention of acute pancreatitis might be important for diabetic patients.

**Abstract:**

The association between acute pancreatitis (AP) and gastrointestinal cancers in diabetic patients is currently not well understood. The study aim was to investigate the association between AP and gastrointestinal cancers in diabetic patients. Data from the Korean National Health Insurance Service database were analyzed. Participants with diabetes who underwent a health examination between 2009 and 2012 were followed up till December 2018. The primary outcome was the occurrence of gastrointestinal cancer. A total of 2,263,184 patients were included in the final analysis. Patients with a history of AP (*n* = 2390) were found to have a significantly higher risk of gastrointestinal cancer, except for esophageal cancer, as follows: gastric cancer (aHR = 1.637, 95% CI: 1.323–2.025), colorectal cancer (aHR = 2.183, 95% CI: 1.899–2.51), liver cancer (aHR = 2.216, 95% CI: 1.874–2.621), pancreatic cancer (aHR = 4.558, 95% CI: 4.078–5.095), bile duct cancer (aHR = 3.996, 95% CI: 3.091–5.269), and gallbladder cancer (aHR = 2.445, 95% CI: 1.459–4.099). The history of AP is associated with the increased risk of gastrointestinal cancer in diabetic patients. It is necessary to investigate the history of AP and more actively recommend screening for gastrointestinal cancers in such patients.

## 1. Introduction

Patients with diabetes have a higher cancer risk than those without diabetes mellitus (DM) [1,2]. Several previous studies have reported the association between type 2 DM and increased risk of cancer at multiple sites including the liver, pancreas, bile duct, gallbladder, colorectum, endometrium, breast, kidney, and bladder [1,3]. The association between type 2 DM and cancer could be caused by biological mechanisms or result from confounding from shared risk factors including obesity, older age, physical inactivity, and smoking [4,5,6]. The molecular pathogenesis of oncogenesis in DM is not completely understood yet. Many researchers have suggested that metabolic dysregulation, hyperglycemia, insulin resistance and subsequent hyperinsulinemia, increased bioactivity of insulin-like growth factor 1, oxidative stress, chronic inflammation, adiposity, altered gut microbiota, dysregulated sex hormones, and genetic factors might play a role in the carcinogenesis in patients with DM [6,7].

Acute pancreatitis (AP) is a common inflammatory disease. Almost all patients with AP have a mild disease. However, some of them have increased morbidity [8]. Late complications of AP including walled-off necrosis that are directly associated with mortality have been well recognized and managed, whereas other long-term outcomes such as AP-related diabetes and cancer remain poorly understood despite their clear association [9,10,11]. The association between AP and pancreatic cancer has been found in a relatively large cohort with a rational explanation [10,11]. However, to the best of our knowledge, how AP affects outcomes of those with other gastrointestinal cancers, especially in diabetic patients, is hardly known.

Therefore, the objective of this nationwide population-based cohort study using National Health Insurance Service (NHIS) data was to investigate the association between AP and malignancy in diabetic patients, focusing on gastrointestinal cancer.

## 2. Materials and Methods

### 2.1. Dataset

The data used in this study were gathered from the NHIS Data Sharing Service of Korea. South Korea has a compulsory national health insurance system. The NHIS covers approximately 97% of Korean population. It provides universal health coverage [12,13]. It provides medical information including patients’ age, sex, living area, insurer payment coverage, deduction and claims data, and medical utilization information. All insured Koreans older than 40 years undergo a biannual health checkup supported by the NHIS. Employees older than 20 years are required to undergo a health checkup once a year. In the NHIS data of health checkup, general health behaviors such as alcohol drinking, smoking, and exercise are surveyed using self-reported questionnaires.

In this study, all personal identification numbers were encrypted, and the need for written informed consent was waived, as the study used de-identified data. None of the patients were contacted. This study was conducted according to the ethical principles outlined in the Declaration of Helsinki. All study procedures and ethical aspects were approved by Soongsil University Institutional Review Board (approval No. SSU-202003-HR-201-01).

### 2.2. Study Population and Design

**Figure 1:**
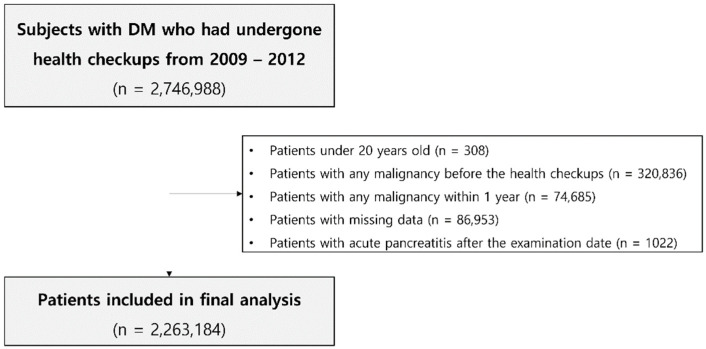
Flowchart showing the selection of subjects for this study.

We used a cohort study design to assess the association of gastrointestinal cancer development with the history of AP in patients with type 2 DM. We included patients diagnosed with type 2 DM who underwent a general health examination from the NHIS data between 2009 to 2012. The general health examination data have changed since 2009 in South Korea, and we searched the data to gather diabetic patients till 2012 in order to secure a sufficient follow-up duration of more than 5 years. Subjects who were under 20 years old, subjects who were diagnosed with any malignancy before or within one year of the lag period after the health checkup date, patients who had missing data, and new cases of AP in participants who had no previous history of AP after the examination date were excluded from the analysis. A total of 2,263,184 patients were finally included (Figure 1). Each person in these retrospective cohorts was followed up for cancer development until 31 December 2018. Those who had no events and were alive were treated as censored data. According to the history of AP, we classified patients into two groups: patients with history of AP (AP group) and patients without history of AP (NAP group).

### 2.3. Predictor, Outcome Variables and Definitions

All diagnoses were determined by combining the 10th edition of the International Statistical Classification of Diseases and Related Health Problems, Clinical Modification (ICD-10-CM) codes, and operational definitions. A diagnosis of type 2 DM was defined as follows: (1) presence of ICD-10-CM codes E11–E14 and claims for at least one oral hypoglycemic agent (OHA) or insulin at baseline, or (2) a fasting glucose level ≥ 126 mg/dL at the general health examination. AP was defined as K85 and hospitalization at the same period. History of AP was defined as having AP within three years prior to the examination date. A history of or newly diagnosed cancer was identified based on the ICD-10-CM code for malignancy (C code) and cancer registration code (V193) in the NHIS data (Table S1). We defined comorbidities as follows. Hypertension was defined as ICD-10-CM codes I10–13 and I15 with antihypertensive medications or systolic blood pressure ≥ 140 mmHg or diastolic blood pressure ≥ 90 mmHg. Dyslipidemia was defined as ICD-10-CM code E78 with antihyperlipidemic medication or total cholesterol ≥ 240 mg/dL. Chronic pancreatitis was defined as ICD-10-CM codes K860, K861, K868, and K903.

Detailed information on patients’ age, sex, general health behavior, socio-demographic variables, and comorbidities were gathered from the NHIS database. We assessed effects of household income at the index date according to two income groups (lowest 20% and the remaining). Subjects were also categorized according to smoking status as never a smoker, former smoker, or current smoker. Alcohol drinking was categorized into none, moderate, or heavy drinkers (≥3 days/week). Regular exercise was defined as mid-intensity exercise ≥ 5 days a week or vigorous exercise ≥ 3 days a week. Operational definitions of study end-points were incidences of cancer.

### 2.4. Statistical Analysis

General characteristics of subjects are presented as means and standard deviation for continuous variables and percentages for categorical variables. Cancer incidence rates were calculated per 1000 person years. The cumulative cancer incidence probability for each cancer was plotted using Kaplan–Meier curves and compared using the log-rank test. Hazard ratios (HRs) and 95% confidence intervals (CIs) for incidence of cancer according to the onset of AP were analyzed using multivariable Cox proportional hazard models with NAP patients as a reference group in crude results. Adjusted HRs were determined after adjusting for age, sex, smoking, alcohol consumption, physical activity, income level, hypertension, dyslipidemia, body mass index (BMI), DM severity, and duration in model 1. They were further evaluated after adjusting for history of chronic pancreatitis in model 2. All statistical analyses were performed using SAS version 9.3 (SAS Institute Inc., Cary, NC, USA). Statistical significance was considered when *p*-value was less than 0.05 in two-tailed *t*-test.

## 3. Results

### 3.1. Baseline Characteristics

Table 1 shows differences in baseline characteristics of the patient population between the AP group (*n* = 2930) and the NAP group (*n* = 2,260,254). The mean age was younger in the AP group. There were more patients aged over 65 years in the NAP group. Both groups showed different distributions of BMI. There were more male patients, current smokers, heavy drinkers, patients with lower income, patients with history of dyslipidemia, insulin users, and patients using more than two OHAs in the AP group. However, there were no significant differences in regular exercise, patients with history of hypertension, or duration of DM between the two groups.

### 3.2. Cancer Incidence According to History of AP in Type 2 DM Patients

Table 2 shows cancer incidence and their differences between the two groups. More patients were diagnosed with any malignancies in the AP group than in the NAP group. All gastrointestinal cancers were more prevalent in the AP group except for gallbladder cancer, which was marginally more prevalent in the AP group. On the other hand, other cancers did not show a significant difference in incidence between the two groups, except that prostate cancer, lung cancer, and laryngeal cancer showed higher incidence in the AP group. Interestingly, ovarian cancer showed a marginally higher incidence in the NAP group.

### 3.3. Cumulative Incidence of Gastrointestinal Cancer According to the History of AP in Type 2 DM Patients

**Figure 2:**
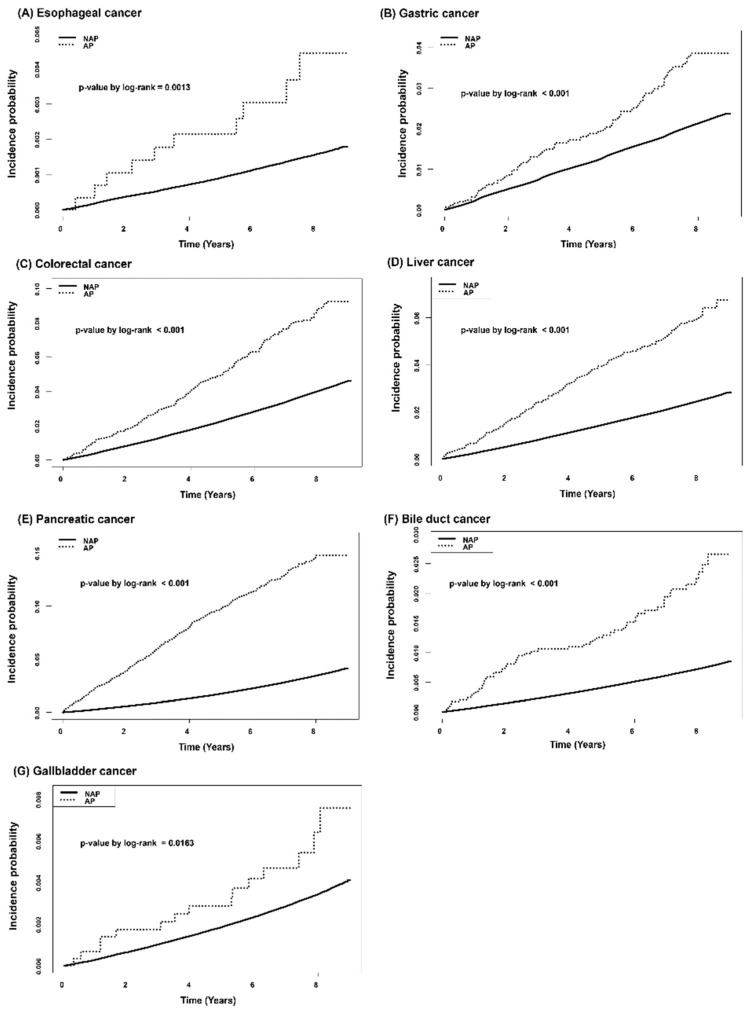
Kaplan-Meier curves for cumulative incidence probability of gastrointestinal cancer according to history of acute pancreatitis: (**A**) Esophageal cancer, (**B**) Gastric cancer, (**C**) Colorectal cancer, (**D**) Liver cancer, (**E**) Pancreatic cancer, (**F**) Bile duct cancer, (**G**) Gallbladder cancer.

Crude incidence rates of all types of gastrointestinal cancers per 1000 person-years were significantly higher in the AP group (Table 3). The cumulative incidence probability for each gastrointestinal cancer according to history of AP was plotted with the Kaplan-Meier survival curve (Figure 2). More patients in the AP group developed all kinds of gastrointestinal cancers, including esophageal cancer, gastric cancer, colorectal cancer, liver cancer, pancreatic cancer, bile duct cancer, and gallbladder cancer. Patients in the AP group were found to have a significantly higher risk for gastrointestinal cancer in multivariable Cox proportional hazard regression models (Table 3). After adjusting for confounding factors in models 1, 2 and 3, risks for all types of gastrointestinal cancer still showed statistical significance except for esophageal cancer.

## 4. Discussion

In this nationwide population-based cohort study, diabetic patients who had a history of AP showed a higher incidence of all gastrointestinal cancers, not only pancreatic cancer. The risk of gastrointestinal cancer in diabetic patients was increased by 1.6 to 4.5 times depending on the history of AP. To the best of our knowledge, this is the first study that evaluates the effect of AP on incidence of gastrointestinal cancers, not only pancreatic cancer.

A recent meta-analysis with bias analysis for unmeasured confounding in 151 cohorts with over 32 million people has reported that cohort-level relative risk for type 2 diabetic patients is 15–25% higher for all-site cancer incidence [2]. Especially, authors of the previous study strongly insist causal associations of type 2 DM with liver and pancreatic cancer incidence. They also suggested possible causal associations between type 2 DM and gallbladder cancer according to their bias analyses. Substantial previous studies have suggested the possible reason for the association between diabetes and increased risk of cancer. Several robust explanations have suggested that cancer and diabetes share potential risk factors common to both and that diabetes may influence the carcinogenesis by several molecular mechanisms, including hyperinsulinemia, hyperglycemia, and chronic inflammation. [3] Therefore, it can be inferred that if diabetes worsens, the risk of cancers in association with diabetes may increase.

A meaningful finding of this study was that a history of AP mainly increased the risk of gastrointestinal cancers in type 2 diabetic patients. This association might be partly explained by shared risk factors between AP and gastrointestinal cancers, such as male gender, alcohol consumption, smoking, and DM [14,15,16,17,18,19,20,21]. These risk factors are associated with metabolic syndrome. A recent large-scale study has reported that metabolic syndrome at baseline is associated with a higher risk of overall gastrointestinal cancer by any definition (HR: 1.21; 95% CI: 1.13–1.29) irrespective of genetic predisposition [22]. Metabolic syndrome may also worsen the prognosis or outcome of AP, as the two interact with each other. An increased risk of gastrointestinal cancer could be affected by history of AP [23]. It has not been clearly proven that the risk of esophageal cancer is increased by DM or metabolic syndrome [2,24]. Results of our study also showed a statistically insignificant association between elevated risk of esophageal cancer and AP in DM patients after adjusting for potential confounders. However, esophageal cancer was associated with AP history significantly in multivariable models without chronic pancreatitis. In addition, excluding other covariates for esophageal cancer in model 3, the significance of HR for esophageal cancer remained statistically significant while chronic pancreatitis is adjusted along with demographic information (age, sex, income level) and lifestyle features (alcohol consumption, smoking, physical activity, BMI). It seems necessary to interpret these results carefully in consideration of overadjustment being affected by lower occurrence [25,26]. On the other hand, there was no increase in the risk according to the history of AP for other types of cancers in this study, such as endometrial cancer, renal cancer, thyroid, leukemia, or breast cancer, known to increase the risk of diabetes in a previous cohort study [2]. Further clinical and basic studies are needed to elucidate the exact effect and mechanism by which AP affects the development of gastrointestinal cancer.

AP is an inflammatory disorder of the pancreas. It is triggered by pathological cellular pathways and organ failures that culminate in acinar cell death and local and systemic inflammation [27]. DM is one of several common adverse events after an episode of AP. The relationship between these two diseases might be quite complicated, as they can affect each other [28]. Recently, a meta-analysis showed that preexisting diabetes had a negative effect on outcomes of AP, as it increased the risk of fatal adverse events [29]. AP might also worsen the course of DM by adversely affecting the pancreas, considering mechanisms of pancreatogenic diabetes including loss of islet cell mass, AP-induced autoimmunity, shared risk factors for AP and diabetes, local and systemic inflammatory response, alterations in the insulin–incretin axis, and a combination of these factors [30]. From this point of view, it can be explained that AP has an additional effect by increasing the risk of cancer in association with DM through mutually negative effects. Epidemiologic studies have provided substantial evidence that AP may lead to the development of pancreatic cancer [10,11,31]. From a traditional point of view, it is a convincing hypothesis that AP occurs repeatedly and progresses to chronic pancreatitis following pancreatic cancer development [32]. However, an epidemiologic study has suggested the potential of an additional pathway from AP to pancreatic cancer [10]. This topic should be investigated further to provide a deeper understanding of not only the relationship of AP with pancreatic cancer but also effects of AP on gastrointestinal cancer.

This study has several strengths. First, there have been few studies about the effect of AP on diabetic patients, especially about long-term outcomes such as cancer risk. Second, this study was based on a huge cohort with a very large number of diabetic patients. Such a large number of patients might compensate for various biases of this study using retrospective claim data. Last, results were obtained after adjusting for various confounding factors of life style and comorbidities.

This retrospective study also has some limitations. First, the results of this study suggest an association rather than a causal relationship. Thus, the results of this study should be interpreted conservatively in consideration of the inherent bias of claim data. In addition, our results were based on operational definitions used commonly in many previous studies. There might be a difference from actual diseases. Second, the results of this study are intuitively understandable. However, it might be difficult to explain the causality or clearly prove it with a controlled study. In addition, the scientific basis for the correlation between AP and gastrointestinal cancer with increased risk was relatively lacking except for pancreatic cancer. Molecular and biological explanations for this part are needed in the future with further basic research. Third, it was difficult to obtain more detailed information about features of AP such as recurrence, etiology, and severity due to limitations of claimed data. When we further conduct subgroup analysis according to two levels of alcohol consumption (heavy drinker vs. non-drinker or moderate drinker), the significant interaction of heavy alcohol consumption was not observed in gastrointestinal malignancies, except for pancreatic cancer (Table S2). However, it is desirable to interpret the subgroup analysis result conservatively, and it seems necessary to conduct further studies in consideration of these clinical characteristics of AP in the future. Lastly, common key risk factors for malignancy such as alcohol consumption and smoking history were defined as categorical variables. Although those risk factors were classified in as much detail as possible, further research with consideration of these key risk factors as continuous variables to evaluate their cumulative effect will be needed.

## 5. Conclusions

In conclusion, a history of AP could particularly increase the risk of gastrointestinal cancer in diabetic patients. It seems necessary to investigate the history of AP in diabetic patients and to regard screening for gastrointestinal cancers in such patients more actively. Results of this study suggest that proper management or prevention of AP might be important for diabetic patients. Further advanced epidemiologic studies, such as a Mendelian randomization study for estimating causal relationships and clinical studies in consideration of detailed features of AP and basic research into molecular mechanisms to evaluate the exact role of AP in the development of cancer in diabetic patients are needed in future to fully understand the increased risk of gastrointestinal cancers in diabetic patients with a history of AP.

## Figures and Tables

**Table 1:** Baseline characteristics of participants according to history of acute pancreatitis.

Variables		AP *n* (%)	NAP *n* (%)	*p*-Value
Total patients		2930	2,260,254	
Age (years)	Mean ± SD	55.33 ± 11.97	56.08 ± 12.51	0.0012
	20–39 years	215 (7.34)	212,649 (9.41)	<0.0001
	40–64 years	2038 (69.56)	1,443,511 (63.86)	
	≥65 years	677 (23.11)	604,094 (26.73)	
Sex	Male	2291 (78.19)	1,356,957 (60.04)	<0.0001
BMI level (kg/m^2^)	<18.5	135 (4.61)	32,546 (1.44)	<0.0001
	18.5–23	1069 (36.48)	552,683 (24.45)	
	23–25	656 (22.39)	560,987 (24.82)	
	25–30	918 (31.33)	936,682 (41.44)	
	30–35	139 (4.74)	156,792 (6.94)	
	≥35	13 (0.44)	20,564 (0.91)	
Smoking	Never	1039 (35.46)	1,244,509 (55.06)	<0.0001
	Former	499 (17.03)	395,058 (17.48)	
	Current	1392 (47.51)	620,687 (27.46)	
Alcohol	None	1433 (48.91)	1,257,753 (55.65)	<0.0001
	Mild	930 (31.74)	767,704 (33.97)	
	Heavy	567 (19.35)	234,797 (10.39)	
Income	Low, 20%	698 (23.82)	436,796 (19.32)	<0.0001
Regular Exercise		594 (20.27)	460,796 (20.39)	0.879
Hypertension		1602 (54.68)	1,226,280 (54.25)	0.647
Dyslipidemia		1219 (41.6)	897,765 (39.72)	0.0372
DM severity	DM over 5 years	840 (28.67)	646,766 (28.61)	0.9483
	Insulin user	891 (30.41)	167,745 (7.42)	<0.0001
	Over two OHA	1356 (46.28)	851,265 (37.66)	<0.0001
Development of chronic pancreatitis during study period		589 (14.81%)	2341 (0.11%)	<0.0001

AP, acute pancreatitis; NAP, no acute pancreatitis; DM, diabetes mellitus; OHA, oral hypoglycemic agents.

**Table 2:** Cancer incidence in diabetic patients according to history of acute pancreatitis.

		AP (*n* = 2930)	NAP (*n* = 2,260,254)	*p*-Value
		*n* (%)	*n* (%)	
Overall		902 (30.78)	415,217 (18.37)	<0.0001
Gastrointestinal cancer	Esophagus	10 (0.34)	3222 (0.14)	0.0044
	Stomach	89 (3.04)	43,882 (1.94)	<0.0001
	Colon and rectum	210 (7.17)	82,274 (3.64)	<0.0001
	Liver	150 (5.12)	50,544 (2.24)	<0.0001
	Pancreas	358 (12.22)	69,453 (3.07)	<0.0001
	Bile duct	56 (1.91)	14,883 (0.66)	<0.0001
	Gallbladder	15 (0.51)	7035 (0.31)	0.0514
Other cancer	Prostate	166 (5.67)	96,315 (4.26)	0.0002
	Lung	109 (3.72)	55,480 (2.45)	<0.0001
	Thyroid	25 (0.85)	24,398 (1.08)	0.2363
	Bladder	23 (0.78)	20,328 (0.9)	0.5122
	Corpus	3 (0.1)	3118 (0.14)	0.6042
	Larynx	12 (0.41)	2211 (0.1)	<0.0001
	Multiple myeloma	12 (0.41)	5869 (0.26)	0.1112
	Kidney	7 (0.24)	10,176 (0.45)	0.0877
	Lymphoma	6 (0.20)	5074 (0.22)	0.8217
	Leukemia	3 (0.1)	3305 (0.15)	0.5348
	Breast	8 (0.27)	10,431 (0.46)	0.1324
	Ovary	7 (0.24)	11,381 (0.5)	0.0431
	Testicle	3 (0.1)	1252 (0.06)	0.2802
	Oral cavity and pharynx	2 (0.07)	1784 (0.08)	0.8371
	Cervix	2 (0.07)	3828 (0.17)	0.1833
	Nerves	6 (0.2)	4470 (0.2)	0.932
	Skin	11 (0.38)	7689 (0.34)	0.7434

AP, patients with history of acute pancreatitis; NAP, patients without history of acute pancreatitis.

**Table 3:** Incidence rates and risk for gastrointestinal cancers in diabetic patients according to history of acute pancreatitis.

**Type**	IR *		**HR for Gastrointestinal Cancer Development in AP Group**			
	AP	NAP	Crude HR (95% CI)	Adjusted HR (95% CI) **	Adjusted HR (95% CI) ***	Adjusted HR (95% CI) ****
Esophagus	0.514	0.195	2.877 (1.547–5.353)	2.079 (1.117–3.869)	1.947 (1.045–3.627)	1.624 (0.852–3.095)
Stomach	4.638	2.677	1.978 (1.606–2.435)	1.770 (1.437–2.179)	1.712 (1.39–2.108)	1.637 (1.323–2.025)
Colon, Rectum	11.169	5.053	2.606 (2.276–2.984)	2.504 (2.187–2.868)	2.367 (2.067–2.711)	2.183 (1.899–2.51)
Liver	7.878	3.084	3.074 (2.619–3.609)	2.760 (2.351–3.24)	2.527 (2.152–2.968)	2.216 (1.874–2.621)
Pancreas	19.705	4.247	6.266 (5.647–6.952)	6.180 (5.569–6.857)	5.659 (5.099–6.281)	4.558 (4.078–5.095)
Bile duct	2.900	0.901	5.135 (3.95–6.676)	4.783 (3.677–6.222)	4.606 (3.539–5.994)	3.996 (3.031–5.269)
Gallbladder	0.771	0.425	2.466 (1.485–4.093)	2.469 (1.487–4.10)	2.415 (1.454–4.012)	2.445 (1.459–4.099)

IR, incidence rate; HR, hazard ratio; AP, acute pancreatitis; NAP, non-acute pancreatitis; CI, confidence interval. * IR was calculated per 1000 person years. ** Adjusted covariates in model 1: age, sex, smoking, alcohol consumption, physical activity, income level, BMI. *** Adjusted covariates in model 2: age, sex, smoking, alcohol consumption, physical activity, income level, hypertension, dyslipidemia, BMI, DM severity and duration. **** Adjusted covariates in model 3: age, sex, smoking, alcohol consumption, physical activity, income level, hypertension, dyslipidemia, chronic pancreatitis, BMI, DM severity and duration.

## Data Availability

The datasets used and/or analyzed in the study are available from the corresponding author on reasonable request.

## Supplementary Materials

The following supporting information can be downloaded at: https://www.mdpi.com/article/10.3390/cancers14225696/s1, Table S1: ICD-10-CM code for each type of cancer used in this study, Table S2: Subgroup analysis and evaluation for interaction according to two level of alcohol consumption.
