# Supplementary material for: Acute Pancreatitis Increases the Risk of Gastrointestinal Cancer in Type 2 Diabetic Patients: A Korean Nationwide Cohort Study
Source: Cancers (Basel). 2022 Nov 20;14(22):5696. doi: 10.3390/cancers14225696 (PMC9688877; doi:10.3390/cancers14225696)
Supplement: Supplementary file 1 [file cancers-14-05696-s001.zip › cancers-2022067-supplementary.pdf]

cancers

MDPI

Supplementary Materials

Acute Pancreatitis Increases the Risk of Gastrointestinal Cancer in Type 2 Diabetic Patients: A Korean Nationwide Cohort Study

Jin Ho Choi, Woo Hyun Paik, Dong Kee Jang, Min Kyu Kim, Ji Kon Ryu, Yong-Tae Kim, Kyungdo Han and Sang Hyub Lee

Table S1. ICD-10-CM code for each type of cancer used in this study.

Cancer Type		ICD-10-CM Code
Overall		C
Gastrointestinal cancers	Esophagus	C15
	Stomach	C16
	Colon and rectum	C18–20
	Liver	C220
	Pancreas	C25
	Biliary cancer	C221, C24
	Gallbladder	C23
Other cancers	Prostate	C61
	Lung	C33, C34
	Thyroid	C73
	Bladder	C67
	Corpus	C54
	Larynx	C32
	Multiple myeloma	C88–90
	Kidney	C64
	Lymphoma	C82–C85
	Leukemia	C91–C95
	Breast	C50
	Ovary	C56
	Testicle	C62
	Oral cavity and pharynx	C00–C06
	Cervix	C53
	Nerves	C71, C72
	Skin	C43, C44

Table S2. Subgroup analysis and evaluation for interaction according to two level of alcohol consumption.

Alcohol consumption level	HR for Gastrointestinal Cancer Development in AP Group vs NAP Group						
	Esophagus	Stomach	Colon and rectum	Liver	Pancreas	Gallbladder	Bile duct
	Crude HR (95% CI)	Crude HR (95% CI)	Crude HR (95% CI)	Crude HR (95% CI)	Crude HR (95% CI)	Crude HR (95% CI)	Crude HR (95% CI)
None or moderate	1.046 (0.25–4.376)	1.688 (1.019–2.728)	2.226 (1.603–3.089)	2.209 (1.571–3.106)	5.58 (4.577–7.554)	0.998 (0.136–7.316)	3.254 (1.558–6.796)
Heavy	1.849 (0.896–3.814)	1.627 (1.285–2.061)	2.167 (1.857–2.53)	2.202 (1.816–2.671)	4.315 (3.808–4.89)	2.733 (1.601–4.665)	4.181 (3.104–5.633)
P for interaction	0.4175	0.9189	0.7197	0.7785	0.0107	0.3355	0.4024

HR, hazard ratio; AP, acute pancreatitis; NAP, non-acute pancreatitis; CI, confidence interval.
